# Supplementary material for: Shiga-Toxin-Producing Strains of Escherichia coli O104:H4 and a Strain of O157:H7, Which Can Cause Human Hemolytic Uremic Syndrome, Differ in Biofilm Formation in the Presence of CO2 and in Their Ability to Grow in a Novel Cell Culture Medium
Source: Microorganisms. 2023 Jul 3;11(7):1744. doi: 10.3390/microorganisms11071744 (PMC10384166; doi:10.3390/microorganisms11071744)
Supplement: Supplementary file 1 [file microorganisms-11-01744-s001.zip › microorganisms-2427746-supplementary.pdf]

**Table S1.** Phenotypic differences displayed by Biolog GEN III plates of *E. coli* strains used in the present study. Only differences between the strains are shown.

| **Compound/Condition** [a] | *E. coli* **Strains** | | | | |
|---|---|---|---|---|---|
| | ATCC 25922 | O157H7 | O104:H4 (Ec 01) (2011C-3493) [b] | O104:H4 (Ec 02) (2009EL-2050)[b] | O104:H4 (Ec 03) (2009EL-2071)[b] |
| Sucrose | — | + | + | + | + |
| D-Raffinose | — | + | + | + | + |
| 8% NaCl | + | — | + | + | + |
| D-Sorbitol | + | — | + | + | + |
| D-Aspartic Acid | — | — | + | + | + |
| D-Serine | + | — | — | — | — |
| Gelatin | — | — | — | — | ± |
| L-Arginine | — | — | — | — | ± |
| D-Saccharic | + | — | — | — | — |
| Naladixic Acid | — | — | + | + | + |
| K Tellurite | — | + | + | + | + |
| β-Hydroxy-DL-Butyric | + | — | — | — | — |
| Propionic Acid | — | + | + | + | + |
| Na Bromate | — | ± | + | — | — |

[a] Symbols represent: +, positive; ±, weak; —, negative.

[b] CDC designation for *E. coli* O104:H4 strains. Ec 01, EC 02, and Ec 03 are designations of the respective Ec strains in this study.

**Table S2.** Comparison of antibiotic susceptibilities between *E. coli* O104:H4 strains and *E. coli* O157:H7.

| Antibiotic | Ec O157:H7 | Ec 01 (2011C-3493) | Ec 02 (2009EL-2050) | Ec 03 (2009EL-2071) |
|---|---|---|---|---|
| | Minimum inhibitory concentration (MIC), μg/ml | | | |
| Trimethoprim | 0.060 | 64.000 | 64.000 | 64.000 |
| Co-Trimoxazole | 0.500 | 64.000 | 64.000 | 64.000 |
| Sulfamethoxazole | 64.000 | 64.000 | 64.000 | 64.000 |
| Azithromycin | 4.000 | 1.000 | 0.125 | 0.125 |
| Doxycycline | 0.500 | 8.000 | 8.000 | 0.250 |
| Tigecycline | 0.060 | 0.060 | 0.060 | 0.060 |
| Cefepime | 0.030 | 64.000 | 0.030 | 0.250 |
| Faropenem | 0.125 | 0.125 | 0.125 | 0.030 |
| Ertapenem | 0.030 | 0.030 | 0.030 | 0.030 |
| Piperacillin | 1.000 | 64.000 | 64.000 | 32.000 |
| Penicillin G | 64.000 | 32.000 | 64.000 | 64.000 |
| Amoxicillin | 2.000 | 64.000 | 64.000 | 64.000 |
| Ceftazidime | 0.250 | 32.000 | 0.030 | 0.125 |
| Imipenem | 0.125 | 0.125 | 0.125 | 0.125 |
| Cefazolin | 2.000 | 64.000 | 2.000 | 4.000 |
| Ampicillin | 0.125 | 32.000 | 32.000 | 32.000 |
| Aztreonam | 0.250 | 32.000 | 0.125 | 0.250 |
| Chloramphenicol | 2.000 | 0.500 | 2.000 | 2.000 |
| Arbekacin | 8.000 | 2.000 | 1.000 | 4.000 |
| Amikacin | 2.000 | 0.250 | 0.500 | 1.000 |
| Gentamicin | 1.000 | 0.125 | 0.250 | 0.250 |
| Netilmicin | 1.000 | 0.250 | 0.060 | 0.125 |
| Streptomycin | 8.000 | 64.000 | 64.000 | 64.000 |
| Ofloxacin | 0.030 | 0.125 | 0.125 | 0.250 |
| Sparfloxacin | 0.030 | 0.030 | 0.030 | 0.030 |
| Moxifloxacin | 0.060 | 0.125 | 0.030 | 0.125 |
| Gemifloxacin | 0.060 | 0.030 | 0.030 | 0.030 |
| Ciprofloxacin | 0.015 | 0.030 | 0.125 | 0.125 |
| Novobiocin | 32.000 | 4.000 | 32.000 | 32.000 |

A. Binding of Congo Red by *E. coli* strains.

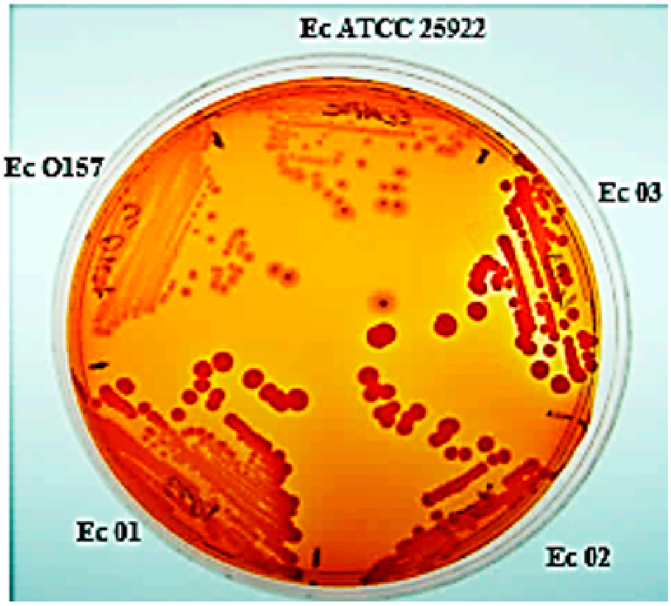


B. Pellets of *E. coli* strains.

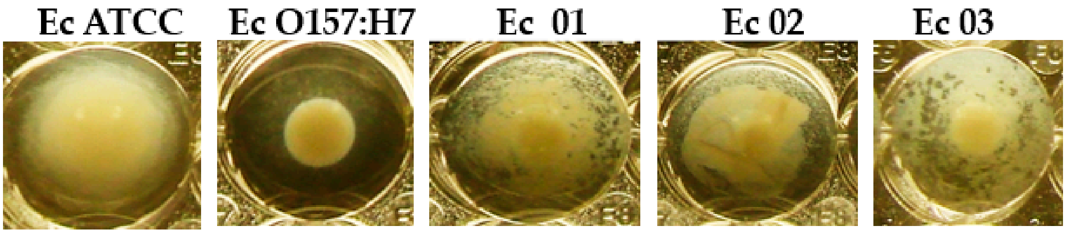


**Figure S1.** Characteristics of *E. coli* strains used in the present study.

**AggA Major pilin**

```
                10         20         30         40         50
Ec01 MKTLKNMRRK NLYITLGLVS LLSGGANAAS QQTTQTIRLT VTNDCPVTIT
Ec02 MKTLKNMRRK NLYITLGLVS LLSGGANAAS QQTTQTIRLT VTNDCPVTIT
Ec03 MKTLKNMRRK NLYITLGLVS LLSGGANAAS QQTTQTIRLT VTNDCPVTIT
                60         70         80         90        100
Ec01 TTPPQTVGVS STTPIGFSAK VTTSDQCIKA GAKVWLWGTG PANKWVLQHA
Ec02 TTPPQTVGVS STTPIGFSAK VTTSDQCIKA GAKVWLWGTG PANKWVLQHA
Ec03 TTPPQTVGVS STTPIGFSAK VTTSDQCIKA GAKVWLWGTG PANKWVLQHA
               110        120        130        140        150
Ec01 KVAKQKYTLN PSIDGGADFV NQGTDAKIYK KLTSGNKFLN ASVSVNPKTQ
Ec02 KVAKQKYTLN PSIDGGADFV NQGTDAKIYK KLTSGNKFLN ASVSVNPKTQ
Ec03 KVAKQKYTLN PSIDGGADFV NQGTDAKIYK KLTSGNKFLN ASVSVNPKTQ
               160     167
Ec01 VLIPGEYTMI LHAAVDF
Ec02 VLIPGEYTMI LHAAVDF
Ec03 VLIPGEYTMI LHAAVDF
```

**AggB Minor pilin**

```
                10         20         30         40         50
Ec01 MLKKSILPMS CGVLVMVMSG LLDAAEITLI SHKTLESQLR DGMKLATGRI
Ec02 MLKKSILPMS CGVLVMVMSG LLDAAEITLI SHKTLESQLR DGMKLATGRI
Ec03 MLKKSILPMS CGVLVMVMSG LLDAAEITLI SHKTLESQLR DGMKLATGRI
                60         70         80         90        100
Ec01 ACREPHNGFY IWINASQNGK VGHYIVQNNR ETKHELKVKI GGGGWSSSLR
Ec02 ACREPHNGFY IWINASQNGK VGHYIVQNNR ETKHELKVKI GGGGWSSSLR
Ec03 ACREPHNGFY IWINASQNGK VGHYIVQNNR ETKHELKVKI GGGGGSSSLR
               110        120        130        140   145
Ec01 EGQRGVYRQG EEKQAIFDIM SDGNQYSAPG EYIFSVSGEC ITSRG
Ec02 EGQRGVYRQG EEKQAIFDIM SDGNQYSAPG EYIFSVSGEC ITSRG
Ec03 EGQRGVYRQG EEKQAIFDIM SDGNQYSAPG EYIFSVSGEC ITSRG
```

AggC Usher protein

```
               10          20          30          40          50
Ec01 MKTSSFIIVI LLCFRIENVI AHTFSFDASL LNHGSGGIDL TLLEKGGQLP
Ec02 MKTSSFIIVI LLCFRIENVI AHTFSFDASL LNHGSGGIDL TLLEKGGQLP
Ec03 MKTSSFIIVI LLCFRIENVI AHTFSFDASL LNHGSGGIDL TLLEKGGQLP
               60          70          80          90         100
Ec01 GIYPVDIILN GSRIDSRDIF FYTKKNRHGE YYLKPCLTRD ILINYGVKIE
Ec02 GIYPVDIILN GSRIDSRDIF FYTKKNRHGE YYLKPCLTRD ILINYGVKIE
Ec03 GIYPVDIILN GSRIDSRDIF FYTKKNRHGE YYLKPCLTRD ILINYGVKIE
              110         120         130         140         150
Ec01 EYPNLFRQNS EKNRDSSDCA DLSVIPQATE DYHFIKQQLI LGIPQVAIHP
Ec02 EYPNLFRQNS EKNRDSSDCA DLSVIPQATE DYHFIKQQLI LGIPQVAIHP
Ec03 EYPNLFRQNS EKNRDSSDCA DLSVIPQATE DYHFIKQQLI LGIPQVAIHP
              160         170         180         190         200
Ec01 PLTGIAHETM WDDGISAFLL NWQVEGGHWE YRSNTRNSSD NFWASLEPGI
Ec02 PLTGIAHETM WDDGISAFLL NWQVEGGHWE YRSNTRNSSD NFWASLEPGI
Ec03 PLTGIAHETM WDDGISAFLL NWQVEGGHWE YRSNTRNSSD NFWASLEPGI
              210         220         230         240         250
Ec01 NLGSWRIRNL TTWNKSSGQS GKWESSYIRV ERGLNNIKSR LTFGDDYTPS
Ec02 NLGSWRIRNL TTWNKSSGQS GKWESSYIRV ERGLNNIKSR LTFGDDYTPS
Ec03 NLGSWRIRNL TTWNKSSGQS GKWESSYIRV ERGLNNIKSR LTFGDDYTPS
              260         270         280         290         300
Ec01 DIFDSVPFRG GMLGSDENMV PYNQREFAPV VRGIARTQAR IEVRQNGYLI
Ec02 DIFDSVPFRG GMLGSDENMV PYNQREFAPV VRGIARTQAR IEVRQNGYLI
Ec03 DIFDSVPFRG GMLGSDENMV PYNQREFAPV VRGIARTQAR IEVRQNGYLI
              310         320         330         340         350
Ec01 QSRIVSPGAF ALTDLPVTGN GGDLQVWVLE SDGTIQTFNV PFTTPAIALR
Ec02 QSRIVSPGAF ALTDLPVTGN GGDLQVWVLE SDGTIQTFNV PFTTPAIALR
Ec03 QSRIVSPGAF ALTDLPVTGN GGDLQVWVLE SDGTIQTFNV PFTTPAIALR
              360         370         380         390         400
Ec01 EGYLKYNVTV GEYRPSDDSI EGAYLGQLTA MYGLPWSLTA FGGIQVSEHY
Ec02 EGYLKYNVTV GEYRPSDDSI EGAYLGQLTA MYGLPWSLTA FGGIQVSEHY
Ec03 EGYLKYNVTV GEYRPSDDSI EGAYLGQLTA MYGLPWSLTA FGGIQVSEHY
              410         420         430         440         450
Ec01 QGNALGLGLS LGGFGSISLD TIYSRGQQKG YSNEIGKTWR VRYDKSFELT
Ec02 QGNALGLGLS LGGFGSISLD TIYSRGQQKG YSNEIGKTWR VRYDKSFELT
Ec03 QGNALGLGLS LGGFGSISLD TIYSRGQQKG YSNEIGKTWR VRYDKSFELT
              460         470         480         490         500
Ec01 GTSFAAGYQD SSAGYHSLAD VLDTYRNGTA YRSYDNRIRR TTINISQALG
Ec02 GTSFAAGYQD SSAGYHSLAD VLDTYRNGTA YRSYDNRIRR TTINISQALG
Ec03 GTSFAAGYQD SSAGYHSLAD VLDTYRNGTA YRSYDNRIRR TTINISQALG
```

```
                510        520        530        540        550
Ec01 EWGSVALNGG RDEYRDKVKQ DYIGASYSNS WKGITFAVNW SRNNNIGDYY
Ec02 EWGSVALNGG RDEYRDKVKQ DYIGASYSNS WKGITFAVNW SRNNNIGDYY
Ec03 EWGSVALNGG RDEYRDKVKQ DYIGASYSNS WKGITFAVNW SRNNNIGDYY
                560        570        580        590        600
Ec01 SNSLRTENNL NLWMSIPMKR WLGGDDKGVT ATAQIQRITG QNTLYETGLN
Ec02 SNSLRTENNL NLWMSIPMKR WLGGDDKGVT ATAQIQRITG QNTLYETGLN
Ec03 SNSLRTENNL NLWMSIPMKR WLGGDDKGVT ATAQIQRITG QNTLYETGLN
                610        620        630        640        650
Ec01 GRAFGQKLYW DIREQIVPGS KYDADTSLLN LRWSGGYGEL TGMYSYNRNT
Ec02 GRAFGQKLYW DIREQIVPGS KYDADTSLLN LRWSGGYGEL TGMYSYNRNT
Ec03 GRAFGQKLYW DIREQIVPGS KYDADTSLLN LRWSGGYGEL TGMYSYNRNT
                660        670        680        690        700
Ec01 RQMNVGTSGS MAIHSGGIAF GQKTDDTMAL IAAPGIAGAS VGGWPGVSTD
Ec02 RQMNVGTSGS MAIHSGGIAF GQKTDDTMAL IAAPGIAGAS VGGWPGVSTD
Ec03 RQMNVGTSGS MAIHSGGIAF GQKTDDTMAL IAAPGIAGAS VGGWPGVSTD
                710        720        730        740        750
Ec01 FRGYTLVGHV SPYQENIITL DPTTFPDNTE VSQTDRRVIP TKGALVQAEF
Ec02 FRGYTLVGHV SPYQENIITL DPTTFPDNTE VSQTDRRVIP TKGALVQAEF
Ec03 FRGYTLVGHV SPYQENIITL DPTTFPDNTE VSQTDRRVIP TKGALVQAEF
                760        770        780        790        800
Ec01 KTRVGNRALV TLTRKDGTLL PFGTVVTLER KTGEAFESAG VVDDKGKVYL
Ec02 KTRVGNRALV TLTRKDGTLL PFGTVVTLER KTGEAFESAG VVDDKGKVYL
Ec03 KTRVGNRALV TLTRKDGTLL PFGTVVTLER KTGEAFESAG VVDDKGKVYL
                810        820        830        840 842
Ec01 SGLSEAGKLK AQWGTNSQCY ADYKLPLKKG MSGIFLTRAV CM
Ec02 SGLSEAGKLK AQWGTNSQCY ADYKLPLKKG MSGIFLTRAV CM
Ec03 SGLSEAGKLK AQWGTNSQCY ADYKLPLKKG MSGIFLTRAV CM
```

AggD Chaperone protein

```
                10         20         30         40         50
Ec01 MKIRRIVSTI AIALSVFTFA HAQSFENVEN NAKVFSLHLG ATRMLYKPNS
Ec02 MKIRRIVSTI AIALSVFTFA HAQSFENVEN NAKVFSLHLG ATRMLYKPNS
Ec03 MKIRRIVSTI AIALSVFTFA HAQSFENVEN NAKVFSLHLG ATRMLYKPNS
                60         70         80         90        100
Ec01 SGETLAVINE HNYPILVQAN VLSEDQKNIA PFIITPPLFR LDALQSSRLR
Ec02 SGETLAVINE HNYPILVQAN VLSEDQKNIA PFIITPPLFR LDALQSSRLR
Ec03 SGETLAVINE HNYPILVQAN VLSEDQKNIA PFIITPPLFR LDALQSSRLR
               110        120        130        140        150
Ec01 IVKTEGAFPI DRESLQWICV KAIPPKYEDK WAKEEVSGKK SDEATMNIQV
Ec02 IVKTEGAFPI DRESLQWICV KAIPPKYEDK WAKEEVSGKK SDEATMNIQV
Ec03 IVKTEGAFPI DRESLQWICV KAIPPKYEDK WAKEEVSGKK SDEATMNIQV

               160        170        180        190        200
Ec01 SVSSCIKLFV RPADVKGQPD DVAGKIKWQK VGNKLKGVNP TPFYMDIAEL
Ec02 SVSSCIKLFV RPADVKGQPD DVAGKIKWQK VGNKLKGVNP TPFYMDIAEL
Ec03 SVSSCIKLFV RPADVKGQPD DVAGKIKWQK VGNKLKGVNP TPFYMDIAEL
               210        220        230        240        250
Ec01 RVGEKEITET HYIAPFSSYE YPMPVNGGGD VRWKVVTDYG GISKTFETGL
Ec02 RVGEKEITET HYIAPFSSYE YPMPVNGGGD VRWKVVTDYG GISKTFETGL
Ec03 RVGEKEITET HYIAPFSSYE YPMPVNGGGD VRWKVVTDYG GISKTFETGL
      252
Ec01 NI
Ec02 NI
Ec03 NI
```

**Figure S2.** Comparison of the amino acid sequences of the AggA, AggB, AggC, and AggD AAF/I proteins from *E. coli* O104:H4 2011C-3493, 2009E-2050, and 2009E-2071 strains.
